# Acquired Hip Dysplasia in Children with Congenital Zika Virus Infection in the First Four Years of Life

**DOI:** 10.3390/v14122643

**Published:** 2022-11-26

**Authors:** Marcos Vinicius da Silva Pone, Tallita Oliveira Gomes da Silva, Carla Trevisan Martins Ribeiro, Elisa Barroso de Aguiar, Pedro Henrique Barros Mendes, Saint Clair dos Santos Gomes Junior, Tatiana Hamanaka, Andrea Araujo Zin, José Paulo Pereira Junior, Maria Elisabeth Lopes Moreira, Karin Nielsen-Saines, Sheila Moura Pone

**Affiliations:** 1 Instituto Nacional de Saúde da Mulher, da Criança e do Adolescente Fernandes Figueira—Fundação Oswaldo Cruz, Rio de Janeiro 22250-020, Brazil; 2 David Geffen School of Medicine, University of California, Los Angeles, CA 90095, USA

**Keywords:** zika virus, zika virus infection, hip dysplasia, hip displacement, hip dislocation

## Abstract

Acquired hip dysplasia has been described in children with cerebral palsy (CP); periodic surveillance is recommended in this population to prevent hip displacement and dislocation. Children with congenital zika syndrome (CZS) may present a spectrum of neurological impairments with changes in tonus, posture, and movement similar to children with CP. However, the relationship between CZS and hip dysplasia has not been characterized. In this prospective cohort study, we aimed to describe the occurrence of hip dysplasia in patients with CZS. Sixty-four children with CZS from 6 to 48 months of age were included and followed at a tertiary referral center in Rio de Janeiro, Brazil, with periodic radiologic and clinical hip assessments. Twenty-six (41%) patients were diagnosed with hip dysplasia during follow-up; mean age at diagnosis was 23 months. According to the Gross Motor Function Classification System (GMFCS), 58 (91%) patients had severe impairment (GMFCS IV and V) at the first evaluation. All patients with progression to hip dysplasia had microcephaly and were classified as GMFCS IV or V. Pain and functional limitation were reported by 22 (84%) caregivers of children with hip dysplasia. All patients were referred to specialized orthopedic care; eight (31%) underwent surgical treatment during follow-up. Our findings highlight the importance of implementing a hip surveillance program and improving access to orthopedic treatment for children with CZS in order to decrease the chances of dysplasia-related complications and improve quality of life.

## 1. Introduction

Zika virus (ZIKV) is an arbovirus identified in Africa in 1947 [1] but only detected in the Americas in 2015 during an exanthematous disease outbreak in Brazil [2,3]. After an increase in the number of newborns with microcephaly [4], a causal relationship between ZIKV infection during pregnancy and the occurrence of fetal malformations was confirmed by epidemiological, clinical and virological data [5,6].

ZIKV exhibits marked neurotropism, impairing central nervous system development and resulting not only in microcephaly but also in a range of neurologic, ophthalmologic and orthopedic abnormalities [7]. Findings related to congenital zika syndrome (CZS) include fetal brain disruption sequence with severe microcephaly, overlapping cranial sutures, prominent occipital bone, redundant scalp skin and neurological impairment; brain anomalies (cerebral cortex thinning, abnormal gyral patterns, increased fluid spaces, corpus callosum anomalies, decreased white matter and cerebellar hypoplasia), especially subcortical calcifications; early pyramidal and extra pyramidal symptoms; ocular anomalies (microphtalmia, coloboma, cataracts and optic nerve hypoplasia), especially chorioretinal atrophy and focal pigmentary mottling affecting the macula; and congenital contractures (clubfoot and arthrogryposis multiplex congenita) [7]. Children with CZS may present with a spectrum of neurological impairments and changes in tonus, posture and movement similar to children with cerebral palsy (CP) [8,9].

Hip dysplasia can be congenital or acquired and is defined as an abnormality in size, morphology or anatomy of the femoral head in the acetabular cavity [10,11]. It can result in subluxation and dislocation of the hip, which may cause pain, functional impairment and difficulty with seating and perineal care [12]. Acquired hip dysplasia and progressive hip displacement occurs in 15 to 30% of children with CP and is related to decreased motor function, spasticity, hypertonia and development delay [13,14,15]. Hip surveillance programs with orthopedic evaluation and routine radiologic assessments are recommended in patients with CP to reduce the incidence of hip dislocation [14,16]. Although well documented in children with CP, the relation between CZS and hip dysplasia has not been characterized. The objective of this study was to describe the occurrence of hip dysplasia in patients with CZS.

## 2. Materials and Methods

This observational study was conducted at the National Institute of Women, Children and Adolescent Health FernandesFigueira (IFF), in Rio de Janeiro, Brazil, a branch of the Oswaldo Cruz Foundation (FIOCRUZ). IFF is the Brazilian Ministry of Health referral center for fetal malformations and pediatric infectious diseases. The institutional review board at IFF approved the study, and all guardians provided written informed consent.

### 2.1. Inclusion and Exclusion Criteria

Patients were referred to our Institute due to confirmed ZIKV maternal infection, with an intrauterine diagnosis of microcephaly or clinical and radiological presentation suggestive of CZS after birth. Children born during and in the aftermath of the ZIKV epidemic (from September 2015 to September 2016) with findings previously described by Moore et al. [7] as characteristics of ZIKV congenital infection were eligible for inclusion in the study, regardless of laboratory confirmation.

We included children from 6 to 48 months of age who had at least two hip radiographic evaluations and two Gross Motor Function Classification System (GMFCS) evaluations during follow-up; children diagnosed with hip dysplasia at the first evaluation, but with no history of congenital hip dysplasia, were also included. Patients with other congenital infections, genetic syndromes, cerebral palsy due to perinatal asphyxia, arthrogryposis or congenital osteoarticular malformations (including congenital hip dysplasia) were excluded.

### 2.2. Study Procedures

We followed patients from March 2016 to July 2019. Pediatric infectious diseases experts documented clinical data, including sex, date of birth, head circumference at birth, child and mother real-time reverse transcription polyermase chain reaction (RT-PCR) result, neurologic assessment, GMFCS classification and whether the child had access to physical therapy consults; caregivers were questioned about pain and functional limitation at every visit. Participants were evaluated by neurologists, pediatric orthopedists and physical therapists.

ZIKV infection was diagnosed using RT-PCR assays with the QuantiTectProbe RT-PCR kit (manufactured by QIAgen, Maryland, MD, USA), using primers and cycles described previously [17], after extraction of RNA with the TRIzolreagent (manufactured by ThermoFisher, Massachusetts, USA), according to the manufacturer’s instructions.

Microcephaly was defined as head circumference at birth −2 (moderate) or −3 (severe) standard deviations (SD) below the mean for gestational age and sex, according to the Intergrowth-21^st^ platform [18]. Motor development and function was assessed using the GMFCS, a five-level classification system for children with CP that is based on self-initiated movement [19,20,21,22]. Each child was evaluated by a Gross Motor Function Measure (GMFM)-trained physical therapist at a minimum of two time points, with the first evaluation after six months of age and follow-up assessments every six months, as part of clinical care. The patient was classified into five progressive levels of independence and functionality according to the GMFCS: level I indicates the ability to walk without any restrictions; level II indicates some limitations in gait; level III indicates children need some assistance to walk; level IV indicates children need assistive technology equipment to move; and level V is reserved for children with severe movement limitations even with the aid of modern technology and total dependence for performance of routine tasks. Children at levels I and II were classified as having mild impairment, at level III as moderate impairment, and at levels IV and V as severe impairment [22,23,24]. Radiological evaluation of the pelvis was performed after six months of age during routine follow-up appointments. Anteroposterior and frog leg lateral hip radiographies were performed by trained radiology technicians with the Siemens Polymatplus S X-ray machine (manufactured by Siemens, São Paulo, Brazil), and evaluated by a trained pediatrician and an orthopedic surgeon. The radiological parameter used to monitor hip displacement is the migration percentage (MP), defined as the percentage of the ossified femoral head outside of the lateral margin of the ossified acetabulum. Hip subluxation or hip displacement defines the state of the hip joint where the MP is between 10% and 99%. Hip dislocation occurs when the femoral head is completely displaced laterally out of the acetabulum (MP = 100%) [13,15].

Children with normal clinical and radiological evaluations had X-rays repeated based on the GMFCS classification. For children classified as GMFCS I, a new evaluation is recommended at the ages of three and five years. Patients classified as GMFCS II should have a new evaluation in one year; for children with GMFCS III to V, a new evaluation should be performed six months after the first screening. Further assessment depends on GMFCS classification changes and MP stability [13,14,15,16]. Patients with hip dislocation were evaluated by an orthopedic surgeon and referred to a specialized center.

### 2.3. Statistical Analysis

Statistical analysis was performed using SPSS software 21.0. We evaluated potential associations between categorical variables using the Chi-square test. *p*-value ≤ 0.05 was considered to be statistically significant.

## 3. Results

Of 108 children with suspected CZS referred to our center, four were diagnosed with genetic syndromes and two with other congenital infections (syphilis and toxoplasmosis); 21 had congenital orthopedic abnormalities and 17 had less than two radiographic evaluations. The remaining 64 children were included in the present study.

Table 1 describes the characteristics of our study population. The median age at the first hip evaluation was 19 months (range 9 to 35 months), and the median age at the last assessment was 32 months (range 24 to 43 months). Eighteen patients were diagnosed with hip dysplasia during the first evaluation; 46 children had two assessments during follow-up, 30 were evaluated three times and 14 were evaluated four times. According to GMFCS assessments, 58 (91%) patients had severe impairment at the first evaluation and 55 (86%) at the last. Hip dysplasia was diagnosed in 26 (41%) children; the median age at diagnosis was 23 months (range 12 to 35 months). Fourteen (54%) of these children were males and twelve (46%) were females; hip alteration was unilateral in 16 patients and bilateral in 10. Twenty-two children with hip dysplasia were classified as GMFCS IV and four as GMFCS V.

All children diagnosed with hip dysplasia had microcephaly; 22 (85%) had severe microcephaly versus 15 (40%) of CZS patients without hip dysplasia. All patients with hip dysplasia had severe impairment according to the GMFCS (classes IV and V) versus 29 (76.5%) patients without hip abnormalities (Table 2). Occurrence of bilateral or unilateral hip dysplasia was not significantly associated with GMFCS classification.

Pain in children with hip dysplasia was reported by 22 of 26 (84%) caregivers, especially during personal hygiene. Functional limitation (inability to perform routine tasks or impaired mobility when seating, walking or even opening legs to change diapers) was also reported in 22 (84%) children at the moment of hip dysplasia diagnosis. Twenty-five (96%) children with hip dysplasia had hypertonia on neurological evaluation; one was hypotonic. Only four (15%) children with hip dysplasia were not routinely undergoing physical therapy at the moment of diagnosis. Eight children were submitted to hip surgery during follow-up.

## 4. Discussion

Previous studies addressing osteoarticular abnormalities in CZS described mainly congenital malformations. Arthrogryposis has been reported in 6 to 11% of children with CZS, and patients with arthrogryposis had a 58 to 100% incidence of congenital hip dislocation [7,25]. The incidence of isolated congenital clubfoot in children with CZS ranges from 4 to 14% [7,25,26]. Children with congenital arthrogryposis and clubfoot were excluded from our sample to avoid confounding with acquired hip dysplasia. The present research is the first to describe acquired hip dysplasia in children with CZS.

A typical hip development results from abnormal contact between the femoral head and the acetabulum. In children with CP, excessive muscle tone and spasticity exert a constant force on the development of the hip, leading to progressive lateral displacement [12]. Delayed motor development, especially in those who do not walk, contributes to progressive hip dysplasia [10].

CZS is a recently recognized congenital infection, and severe motor and neurological impairment have been well-described [27]. Previous studies indicate that hyperreflexia occurs in 100% of children with CZS, hypertonicity in 95% and clonus in 77% [28]. Motor function impairment in CZS can cause muscle shortening and reduce hip mobility [28]. Occurrence of acquired hip dysplasia was therefore expected and is confirmed by our data. In the present study, the frequency of hip dysplasia among children with CZS was 41%; previous studies analyzing children with other causes of CP found an overall risk of 15–30% [13,14,15,16]. In our study, all patients with hip dysplasia had microcephaly, with 85% being severe and the vast majority being hypertonic. Smaller head circumferences have been associated with greater brain damage and greater motor impairment [29].

In the general population and in patients with CP, hip dysplasia is more frequent in females due to ligament laxity [30]. In our study, there was a slight predominance of males with hip dysplasia (54%), but this might be due to the fact that 61% of the 64 children enrolled in the study were male. Children with CP of other etiologies more frequently present left hip dysplasia [30]; in our study, however, right or bilateral involvement were more frequent.

Hip surveillance is effective in preventing hip displacement and reducing pain and movement limitation in children with CP and should be carried out in all children with this condition, regardless of functional evaluation [12,13,14,15,16,31]. Published guidelines recommend that children with spasticity, diplegia or quadriplegia should have their first hip radiograph between the first and second year of life [13,15,30]. Periodic assessments are then recommended based on GMFCS and MP [12,13,15]. Children with CP are at a higher risk of hip dislocation after four years of age [12]. In the present study, the first radiological evaluation occurred on average at 20 months, with 69% of patients diagnosed with hip dysplasia in their first evaluation. However, adequate hip surveillance was a challenge due to loss of follow-up, missed appointments or hospitalizations, with 15% of children having only one or two evaluations.

In addition to radiological surveillance, hip dislocation can be suspected when there is gait abnormality, limitation in hip abduction movement or pain during manipulation. Pain is usually reported by 60% of caregivers of children with CP, but its identification presents a challenge due to impaired verbal communication. Frequent night awakenings, irritability and crying when moving, and especially when changing diapers, may represent indirect signs of pain [31,32,33]. In our study, caregivers reported pain in 84% of children with dysplasia, especially during diaper change. This basic routine care requires abduction of the lower limbs; during this movement, the femoral head may slide out of the acetabular margin, due to the adductor tension, causing pain [31,32].

Among children with CP who develop hip dysplasia, patients with spastic quadriplegia have a higher risk (75%) when compared with those with spastic hemiplegia or ataxia and diplegia or dystonia [30]. Functional limitation leads to decreased range of motion and is also associated with progression to hip dysplasia [13,15]. In previous studies, most children with CZS presented with spastic quadriplegia [30]. The majority ofchildren with hip dysplasia in our cohort (84%) had significant functional limitation at the time of diagnosis; only 8% of children were able to walk by the end of the study. These findings reinforce the need for hip surveillance in this group of patients.

A linear relationship between hip dislocation and the functional level and gross motor function (expressed through GMFCS) of the child with CP has been clearly described [12,13,14,15,16,34]. Children with GMFCS I and II have a very low risk of dysplasia (5%), while children classified as IV and V have a risk of 68% to 90% [15].In our study, only children with GMFCS IV and V progressed to hip dysplasia, with more than half and about one-fifth of children with GMFCS IV and GMFCS V, respectively, developing hip dysplasiain the first four years of life. However, the GMFCS evaluation is more reliable after two years of age [21,22], while in our population, the median age at diagnosis was 23 months. Therefore, the functional classification of these patients can still change.

Prevention of progressive hip dislocation involves physical therapy, including weight loading on the hip, which facilitates the fitting between the femoral head and the acetabulum [34,35]. In our study, there was no association between occurrence of hip dislocation and motor physical therapy; even children who received regular physical therapy presented hip dysplasia. However, we did not assess frequency, duration, regularity, and quality of physical therapy, which was a study limitation.

Surgical management aims to prevent pain and maintain hip flexibility and a symmetrical range of motion; its indication depends on the degree of displacement of the femoral head from the acetabulum [12,16,32,35]. Early orthopedic intervention reduces the need for reconstructive surgeries and avoids the performance of salvage surgery [16,31]. The goal of hip surveillance is timely referral for orthopedic evaluation; timely intervention can prevent hip deterioration and improve the quality of life [15,34]. In our study, all children diagnosed with hip dysplasia were eligible for surgery and referred to orthopedic care in the public health system. However, access to an orthopedic service and to surgical treatment was often delayed, and only 31% of children underwent surgical treatment.

Even though hip dysplasia has been well documented in children with CP, to our knowledge, this is the first study to describe the relationship between this orthopedic condition and CZS. Despite challenges related to missed visits, we performed a prospective and longitudinal assessment including radiological and functional evaluation in a large number of children with CZS.

## 5. Conclusions

Children with CZS have a high risk of hip dysplasia, associated with serious neurological impairment and severe functional limitation. Unilateral right or bilateral involvement were more frequent in our cohort, while the diagnosis was often made before two years of age. Our results reinforce the importance of implementing a hip surveillance program in patients with CZS and improving access to orthopedic treatment. Early identification is essential for an appropriate therapeutic approach, decreasing the chances of dysplasia-related complications and helping improve the quality of life of these children, thus allowing them to achieve stable, mobile, and painless hips [34].

## Figures and Tables

**Table 1:** Description of the study population (total=64).

	N (%)
**Sex**	
Male	39 (61%)
**Head circumference**	
Normal	4 (6%)
Microcephaly	60 (94%)
Severe	37 (58%)
Moderate	23 (36%)
**Child ZIKV RT-PCR result**	
Positive	11 (17%)
Negative	19 (30%)
Not performed	34 (53%)
**GMFCS classification—first assessment**	
I	0
II	4 (6%)
III	2 (3%)
IV	40 (63%)
V	18 (28%)
**GMFCS classification—last assessment**	
I	4 (6%)
II	1 (2%)
III	4 (6%)
IV	47 (73.5%)
V	8 (12.5%)
**Hip dysplasia**	
Yes	26 (41%)
Unilateral	16 (25%)
Right	11 (17%)
Left	5 (8%)
Bilateral	10 (16%)
No	38 (59%)
**Moment of diagnosis of hipdysplasia**	
First evaluation	18 (69%)
Second evaluation	5 (19%)
Third evaluation	2 (8%)
Fourth evaluation	1 (4%)

GMFCS: Gross Motor Function Classification System; RT-PCR: real-time polymerase chain reaction; ZIKV: zika virus.

**Table 2:** (**A**) Frequency of hip dysplasia in children with and without microcephaly. (**B**) Frequency of hip dysplasia in children according to GMFCS classification.

**(A)**			**Microcephaly**		
			**Yes (N = 60)**		**No (N = 4)**
			**Moderate (N = 23)**	**Severe (N = 37)**	
**Hip dysplasia**		**Yes**	4 (17%)	22 (59%)	0
		**No**	19 (83%)	15 (31%)	4 (100%)
**(B)**			GMFCS *		
			**I to II (mild) (N = 5)**	**III (moderate) (N = 5)**	**IV and V (severe) (N = 55)**
**Hip dysplasia**	**Yes**		0	0	26 (47%)
	**No**		5 (100%)	4 (100%)	29 (53%)

* GMFCS: Gross Motor Function Classification System.

## Data Availability

Not applicable.
